# Supplementary material for: An open-source framework for large-scale, flexible evaluation of biomedical text mining systems
Source: J Biomed Discov Collab. 2008 Jan 29;3:1. doi: 10.1186/1747-5333-3-1 (PMC2276192; doi:10.1186/1747-5333-3-1)
Supplement: Additional file 1 — Evaluation platform source code. The additional file contains the software evaluation framework discussed in this paper. The most current release can be downloaded from SourceForge.net [1]. See the accompanying README file for instructions on installation and use. Also included in the distribution is a collection of UIMA wrappers for some commonly used BioNLP tools and annotated corpora. [file 1747-5333-3-1-S1.zip › bionlp_uima_repository-1.0.1/docs/api/src-html/edu/uchsc/ccp/uima/ae/nlp/ei/mutation/MutationFinder_AE.html]

```
001    /*
002     * Created on Sep 8, 2005
003     *
004     * TODO To change the template for this generated file go to
005     * Window - Preferences - Java - Code Style - Code Templates
006     */
007    package edu.uchsc.ccp.uima.ae.nlp.ei.mutation;
008    
009    import java.util.Map;
010    import java.util.Set;
011    
012    import org.apache.uima.analysis_engine.ResultSpecification;
013    import org.apache.uima.analysis_engine.annotator.AnnotatorConfigurationException;
014    import org.apache.uima.analysis_engine.annotator.AnnotatorContext;
015    import org.apache.uima.analysis_engine.annotator.AnnotatorContextException;
016    import org.apache.uima.analysis_engine.annotator.AnnotatorInitializationException;
017    import org.apache.uima.analysis_engine.annotator.AnnotatorProcessException;
018    import org.apache.uima.analysis_engine.annotator.JTextAnnotator_ImplBase;
019    import org.apache.uima.cas.CASException;
020    import org.apache.uima.jcas.JCas;
021    import org.apache.uima.jcas.cas.FSArray;
022    
023    import edu.uchsc.ccp.nlp.ei.mutation.Mutation;
024    import edu.uchsc.ccp.nlp.ei.mutation.MutationException;
025    import edu.uchsc.ccp.nlp.ei.mutation.MutationFinder;
026    import edu.uchsc.ccp.nlp.ei.mutation.PointMutation;
027    import edu.uchsc.ccp.uima.annotation.CCPAnnotationSet;
028    import edu.uchsc.ccp.uima.annotation.CCPAnnotator;
029    import edu.uchsc.ccp.uima.annotation.CCPSpan;
030    import edu.uchsc.ccp.uima.annotation.CCPTextAnnotation;
031    import edu.uchsc.ccp.uima.mention.CCPClassMention;
032    import edu.uchsc.ccp.uima.util.UIMA_Util;
033    
034    /**
035     * @author williamb Takes longer time to run the first time.. not sure why
036     */
037    public class MutationFinder_AE extends JTextAnnotator_ImplBase {
038        private final boolean DEBUG = false;
039    
040    private MutationFinder mutationFinder;
041    
042        /**
043         */
044        public static final String PARAM_REGEX_FILE = "RegExFile";
045    
046        public void initialize(AnnotatorContext ac) throws AnnotatorInitializationException, AnnotatorConfigurationException {
047    
048            
049            try {
050                String regexFile =  (String) ac.getConfigParameterValue(PARAM_REGEX_FILE);
051                mutationFinder = new MutationFinder(regexFile);
052            } catch (AnnotatorContextException ace) {
053                ace.printStackTrace();
054            }
055            
056            
057            super.initialize(ac);
058        }
059    
060        public void process(JCas jcas, ResultSpecification arg1) throws AnnotatorProcessException {
061           
062            try {
063                Map<Mutation, Set<int[]>> mutation2spanMap = mutationFinder.extractMutations(jcas.getDocumentText());
064                
065                /* for each mutation found, create an annotation */
066                for (Mutation mutation : mutation2spanMap.keySet()) {
067                    if (mutation instanceof PointMutation) {
068                        PointMutation pointMutation = (PointMutation) mutation;
069                        Set<int[]> spans = mutation2spanMap.get(mutation);
070                        for (int[] span : spans) {
071                            createPointMutationAnnotation(pointMutation, span, jcas);
072                        }
073                    }
074                }
075                
076            } catch (MutationException e) {
077                e.printStackTrace();
078            }
079        }
080    
081        private void createPointMutationAnnotation(PointMutation pointMutation, int[] span, JCas jcas) {
082    //        System.err.println("Creating mutation mention: " + pointMutation.toString());
083            
084            CCPTextAnnotation mutAnnot = new CCPTextAnnotation(jcas);
085    
086            char mut = pointMutation.getMutResidue();
087            char wt = pointMutation.getWtResidue();
088            int pos = pointMutation.getPosition();
089            
090            int startIndex = span[0];
091            int endIndex = span[1];
092            
093            mutAnnot.setBegin(startIndex);
094            mutAnnot.setEnd(endIndex);
095    
096            /* create span */
097            FSArray ccpSpans = new FSArray(jcas, 1);
098            CCPSpan ccpSpan = new CCPSpan(jcas);
099            ccpSpan.setSpanStart(startIndex);
100            ccpSpan.setSpanEnd(endIndex);
101            ccpSpans.set(0, ccpSpan);
102            mutAnnot.setSpans(ccpSpans);
103    
104            /* set annotator and annotation set */
105            CCPAnnotator ccpAnnotator = new CCPAnnotator(jcas);
106            ccpAnnotator.setAffiliation("UCHSC");
107            ccpAnnotator.setFirstName("MutationFinder");
108            ccpAnnotator.setAnnotatorID(77);
109            ccpAnnotator.setLastName("MutationFinder");
110    
111            CCPAnnotationSet ccpAnnotationSet = new CCPAnnotationSet(jcas);
112            ccpAnnotationSet.setAnnotationSetID(-1);
113            ccpAnnotationSet.setAnnotationSetName("Default Set");
114            ccpAnnotationSet.setAnnotationSetDescription("");
115    
116            mutAnnot.setAnnotator(ccpAnnotator);
117            FSArray asets = new FSArray(jcas, 1);
118            asets.set(0, ccpAnnotationSet);
119            mutAnnot.setAnnotationSets(asets);
120    
121            mutAnnot.setDocumentSectionID(-1);
122    
123            CCPClassMention ccpCM = new CCPClassMention(jcas);
124            ccpCM.setMentionName("mutation");
125            
126            try {
127                UIMA_Util.addSlotValue(ccpCM, "wildtype residue", Character.toString(wt));
128                UIMA_Util.addSlotValue(ccpCM, "mutant residue", Character.toString(mut));
129                UIMA_Util.addSlotValue(ccpCM, "position", Integer.toString(pos));
130            } catch (CASException e1) {
131                e1.printStackTrace();
132            }
133            
134            mutAnnot.setClassMention(ccpCM);
135            try {
136                UIMA_Util.addCCPTextAnnotationToCCPClassMention(mutAnnot, ccpCM);
137            } catch (CASException e) {
138                e.printStackTrace();
139            }
140            mutAnnot.addToIndexes();
141            
142    //        if ((numIDs > 0) && (numIDs < 10)) {
143    //            consensusProtein.addToIndexes();
144    //        }
145    
146        }
147    }
```
